# Association of Pain Phenotypes with Risk of Falls and Incident Fractures

**DOI:** 10.3390/biomedicines10112924

**Published:** 2022-11-14

**Authors:** Maxim Devine, Canchen Ma, Jing Tian, Benny Antony, Flavia Cicuttini, Graeme Jones, Feng Pan

**Affiliations:** 1 Menzies Institute for Medical Research, University of Tasmania, Hobart 7000, Australia; 2 Department of Epidemiology and Preventive Medicine, Monash University Medical School, Commercial Road, Melbourne 3181, Australia

**Keywords:** falls risk, incident fractures, pain phenotypes

## Abstract

**Objective:** To compare whether falls risk score and incident fracture over 10.7 years were different among three previously identified pain phenotypes. **Methods:** Data on 915 participants (mean age 63 years) from a population-based cohort study were studied at baseline and follow-ups at 2.6, 5.1 and 10.7 years. Three pain phenotypes were previously identified using the latent class analysis: Class 1: high prevalence of emotional problems and low prevalence of structural damage; Class 2: high prevalence of structural damage and low prevalence of emotional problems; Class 3: low prevalence of emotional problems and low prevalence of structural damage. Fractures were self-reported and falls risk score was measured using the Physiological Profile Assessment. Generalized estimating equations model and linear mixed-effects model were used to compare differences in incident fractures and falls risk score over 10.7 years between pain phenotypes, respectively. **Results:** There were 3 new hip, 19 vertebral, and 121 non-vertebral fractures, and 138 any site fractures during 10.7-year follow-up. Compared with Class 3, Class 1 had a higher risk of vertebral (relative risk (RR) = 2.44, 95% CI: 1.22–4.91), non-vertebral fractures (RR = 1.20, 95% CI: 1.01–1.42), and any site fractures (RR = 1.24, 95% CI: 1.04–1.46) after controlling for covariates, bone mineral density and falls risk score. Class 2 had a higher risk of non-vertebral and any site fracture relative to those in Class 3 (non-vertebral: RR = 1.41, 95% CI: 1.17–1.71; any site: RR = 1.44, 95% CI: 1.20–1.73), but not vertebral fracture. Compared with Class 3, Class 1 had a higher falls risk score at baseline (β = 0.16, 95% CI: 0.09–0.23) and over 10.7-year (β = 0.03, 95% CI: 0.01–0.04). **Conclusions:** Class 1 and/or Class 2 had a higher risk of incident fractures and falls risk score than Class 3, highlighting that targeted preventive strategies for fractures and falls are needed in pain population.

## 1. Introduction

Musculoskeletal pain is common in older adults with a prevalence ranging from 61% to 74% [1,2] and causes a significant burden on both individual and societal healthcare. The 2016 Global Burden of Disease Study has reported low back and neck pain ranking as 1st and 6th causes of years lived with disability among 30 leading diseases and injuries [3]. The burden of musculoskeletal pain and related health outcomes has been projected to rise continually by reducing physical function and quality of life, increasing rates of disability, and developing mortality in older adults [4,5].

As the major symptom of osteoarthritis (OA), musculoskeletal pain is highly heterogenous and affected by multiple factors, including peripheral, psychological, and neurological factors [6]. Studies have shown that 20% of musculoskeletal pain is ascribed to OA [7]. The heterogeneity of pain imposes difficulty in the effective intervention of pain conditions. Indeed, the “one-size-fits-all” approach may overlook the heterogeneity of pain and interactions between pain-related factors. Thus, identification of pain subgroups has been suggested as a novel approach to tailored pain management and decision-making for preventing related clinical outcomes [8]. Observational studies have demonstrated different health-related quality of life, disease activity, and mortality risk across musculoskeletal pain subgroups/phenotypes [8,9,10]. 

Fracture is a critical healthcare issue worldwide, resulting in recurrent fractures and subsequent mortality [11,12,13]. Ageing, osteoporosis, and falls are the major risk factors for fractures [14,15]. Musculoskeletal pain has been linked to an increased risk of falls, due to local joint pathology, muscle weakness, reduced neuromuscular response and slowed cognition, greater difficulty mobilising [16,17,18,19]. Pain has been suggested as an independent risk factor for fractures, although the results were contradictory. It has been proposed that inflammation related to pain is likely to play a role in the process of bone remodelling and thereby increasing the risk of fractures [20,21]. A previous large cohort study reported an association between severity of knee pain and non-vertebral and hip fractures, and our recent study demonstrated a dose-manner relationship between the number of painful sites and increased risk of fractures at both vertebral and non-vertebral sites [22,23]. In contrast, no associations between pain and incident hip or non-vertebral fractures were observed in men aged over 65 years with a 9.7-year follow-up time from a large multicentre prospective study [24]. 

Our recent study has identified three distinctive knee pain phenotypes by considering a broad range of pain-related factors, including structural abnormalities on magnetic resonance imaging (MRI), emotional issues, body mass index (BMI): Class 1: high prevalence of emotional problems and low prevalence of structural damage (38%); Class 2: high prevalence of structural damage and low prevalence of emotional problems (17%); Class 3: low prevalence of emotional problems and low prevalence of structural damage (45%) [25,26]. Further, pain severity and number of painful sites were found to be different between the classes/subgroups [26]. Given the link between pain and an increased risk of falls and fracture and the heterogeneity of pain, we hypothesised that the risk of falls and/or incident fractures was phenotype-specific in pain population. Therefore, this study was to compare whether falls and incident fractures risk over 10.7 years were different among the three knee pain phenotypes we previously identified. 

## 2. Method

### 2.1. Participants 

This study was conducted as part of the Tasmanian Older Adult Cohort Study (TASOAC). Participants aged 50–80 years [median (interquartile range), 62 (57–69) years] were randomly selected from the electoral roll in Southern Tasmania (43° S, southern part of island state in Australia, population 229,000), using sex-stratified random sampling. Participants were studied at baseline (*n* = 1099), 2.6 (*n* = 875), 5.1 (*n* = 768) and 10.7 (*n* = 563) years. The current study consisted of 915 participants who had been identified pain phenotypes and had complete data on interview and general questionnaires, bone mineral density (BMD), falls, and fractures. The study was approved by the Southern Tasmanian Health and Medical Human Research Ethics Committee (Ref. No: H0006488), and written informed consent was obtained from all participants.

### 2.2. Measurements for Factors to Identify Pain Phenotypes

Measurements of knee structural abnormalities on MRI, emotional problems, number of painful sites, BMI, sex, education level, and comorbidities, which were measured by trained observer(s) or self-report questionnaires at baseline, were used to identify pain phenotypes. The details of each measurement have been described elsewhere [25,26]. In brief, each participant had an MRI scan on their right knee in the sagittal plane on a 1.5-T whole body MR unit (Picker, OH) using a commercial transmit–receive extremity coil. The sequences used have been previously described [27]. Cartilage defects, bone marrow lesions (BMLs), and effusion-synovitis were assessed on MR images at the medial tibial, medial femoral, lateral tibial, and lateral femoral sites. Emotional problems were assessed by using one single mental health item from the short form-8 [28]. Participants reported whether they had pain (yes/no) occurring at their neck, back, hands, shoulders, hips, knees, or feet. A total number of painful sites was created by summing each site (ranging 0–7). Weight and height were measured, then BMI was calculated (kg/m^2^). Sex was collected during interview. Highest education level was self-reported and grouped into three categories (low, medium, high). Common conditions including diabetes, myocardial infarction, hypertension, thrombosis, asthma, bronchitis/emphysema, osteoporosis, hyperthyroidism, hypothyroidism, and rheumatoid arthritis were recorded using a self-reported comorbidity questionnaire. Heart attack, hypertension, diabetes and rheumatoid arthritis have been reported to be linked to musculoskeletal pain [29]. Therefore, the presence of comorbidity was defined as participants having any of these four comorbidities.

### 2.3. Measurements for Outcomes

#### 2.3.1. Incident Fractures

Fractures were self-reported at baseline, approximately 2.6-, 5- and 10.7-year follow-up. Participants responded to the following question: “List any fracture you may have had since your previous interview for this study. Please list these by the location of the fractures (e.g., left thumb, right wrist)” [30]. Incident fractures were classified as non-vertebral, vertebral, and any site fractures. 

#### 2.3.2. Falls Risk Score

Falls risk score was estimated from the physical profile assessment (PPA) at each time-point [31]. Performance in five physiological domains, including knee extension strength, balance, proprioception, reaction time, and edge contrast sensitivity, was assessed to calculate standardized Z-score for falls risk [31]. 

### 2.4. Measurements for Other Related Factors

At baseline, PA was measured by steps per day over seven consecutive days using a pedometer (Omron HJ-003 & HJ-102; Omron Healthcare, Kyoto, Japan). Our criteria for the inclusion of pedometer estimates have been described previously [32]. Hip BMD was measured by the dual-energy X-ray absorptiometry (Hologic, Waltham, MA, USA). The Hologic densitometer was calibrated automatically using the internal software system [33]. Age, smoking history, and pain medication use were recorded via a questionnaire at baseline. 

### 2.5. Statistical Analysis

#### 2.5.1. Identifying Pain Phenotypes

Methods for identifying pain phenotypes in this cohort have been described in detail [25,26]. Briefly, latent class analysis (LCA) was applied to identify groups of participants with similar profiles according to their baseline characteristics related to pain (i.e., sex, BMI, emotional problems, education level, comorbidities, number of painful sites, and MRI-detected knee structural damage). Three knee pain phenotypes were identified: Class 1: high prevalence of emotional problems and low prevalence of structural damage (38%); Class 2: high prevalence of structural damage and low prevalence of emotional problems (17%); Class 3: low prevalence of emotional problems and low prevalence of structural damage (45%). Pain severity in Class 1 and Class 2 was greater than that in Class 3.

#### 2.5.2. Comparing Risks of Falls and Incident Fractures over 10.7 Years across Three Knee Pain Phenotypes

The characteristics of participants were compared across the pain phenotypes identified from the LCA using analysis of variance or multi-nominal logistic regression.

Falls risk score was normally distributed; therefore, linear mixed-effects model with a fixed effect for age, physical activity, smoking history, pain medication use at baseline, and random intercepts for follow-ups was used to compare the differences in falls risk score over 10.7-year between pain phenotypes. In the same mixed-effects model, the interaction term of pain phenotypes and follow-ups was used to compare the differences in change of falls risk score over 10.7-year between pain phenotypes.

Generalized estimating equations (GEE) log-binomial models with robust standard errors and adjustment for age, physical activity, smoking history, pain medication use, falls risk score, and hip BMD at baseline were used to compare the differences in incident fractures between pain phenotypes. 

Stata V.15 was used for the analyses, and LCA analysis was performed using LCA Stata Plugin [34]. *p* values less than 0.05 (two-tailed) were regarded as statistically significant.

## 3. Results

The characteristics of the participants within each class are shown in Table 1. Classes 1, 2, 3 consisted of 38%, 17%, 45% of participants in this study. Compared with Classes 2 and 3, participants in Class 1 were more likely to be female, had lower hip BMD. Participants in Class 2 were older, more likely to be male, had higher BMI, and higher hip BMD than participants in Class1 and 3. Participants in Class 3 tended to have lower BMI than those in Class 1 and 2. There were 3 new hip, 19 vertebral, and 121 non-vertebral fractures, and 138 any site fractures during 10.7-year follow-up. Participants in Class 1 had higher incidences of non-vertebral and any site fractures than Class 3. 

**Figure 1:**
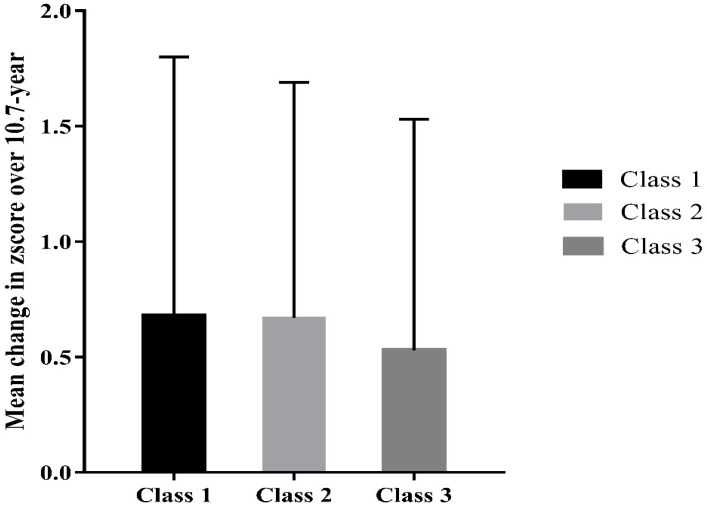
Mean change of falls risk score over 10.7-year in each class.

Figure 1 shows changes in falls risk score over 10.7-year. Participants in Class 1 had a larger increase in falls risk score compared with Class 2 and 3 (Figure 1). When comparing the differences in falls risk score over 10.7 years between classes, participants in Class 1 had a higher falls risk score relative to those in Class 2 and 3 (Table 2). Participants in Class 2 had a lower falls risk score than those in Class 3. There was a greater change in falls risk score in Class 1 compared with Class 3. Changes in the falls risk score were not different between Class 1 and 2 and between Class 2 and 3.

Table 3 shows the differences in incident fracture risks between classes after controlling for covariates, BMD, and falls risk score. Compared with Class 3, participants in Class 1 had a higher risk of vertebral, non-vertebral, and any site fractures. Participants in Class 2 had a higher risk of non-vertebral and any site fractures than those in Class 3, but not vertebral fracture. There were no differences in risks of vertebral, non-vertebral and any site fractures between Class 1 and Class 2.

## 4. Discussion

This study found that falls risk score and risks of incident fracture differ across the three knee pain phenotypes. Class 1 (high prevalence emotional problems/low prevalence structural damage) was associated with a higher risk of falls compared to Class 2 (high prevalence structural damage/low prevalence emotional problems), and Class 3 (low prevalence emotional problems/low prevalence of structural damage). Participants in Classes 1 and 2 suffered from greater pain severity than those in Class 3, had a higher risk of incident fractures than those in Class 3, independent of falls risk score, BMD and covariates. These findings suggest that falls risk score and risks of incident fractures are manifested differently across pain phenotypes, and highlight that targeted preventive strategy for fractures and falls is needed in a specific pain phenotype. 

Pain and the risk of falls have been suggested to have a link [16,17,35,36,37,38,39] due to joint pathology, muscle weakness, or slowed neuromuscular responses and cognitive/executive function [18,19]. The current study found a higher falls risk score in Class 1, compared with Classes 2 and 3. In addition, Class 1 had a greater change in falls risk score over 10.7 years, compared with Class 3. One of explanations for a greater falls risk score observed in Class 1 may be due to a greater pain severity in Class 1 at each time-point compared with Classes 2 and 3. This is supported by a 4-year follow-up cohort study including 765 participants aged over 70 years, which reported that presence of moderate-to-severe pain (yes/no) was associated with an increased risk of falls occurrence [40]. Similarly, a cross-sectional study from Framingham of older adults with a mean age of 69 years showed that severity of pain in foot was associated with a higher risk of falls occurrence [41]. However, we found that participants in Class 2 with distinctive structural damages had a lower falls risk score than Class 3, although Class 2 had a greater pain score than Class 3. This suggests that falls risk is not fully determined by pain itself but mediated by other factors. A recent study with compelling data reported that concerns about falls, knee strength, and standing balance were mediators of the relationship between knee pain and multiple falls [42]. The current study also found that the knee pain population with more emotional problems rather than structural damages was more likely to have a higher falls risk score. The plausible mechanisms between psychological problems and falls risk involve low self-efficacy, executive dysfunction, gait change, impairment of balance performance [43]. These findings suggest that psychological problems are more likely to drive the falls than pain resulting from structural damages. Intervention for psychological health in pain population may be effective in reducing falls risk.

Falls risk has been suggested as a major cause of fractures, but an increased risk of falls cannot fully explain relationships between pain and fractures. Indeed, our results found that different fractures risk were observed in different pain phenotypes independent of falls risk, suggesting that relationships between pain phenotypes and risk of fractures may not be mediated by pain-related falls risk. In line with a 3-year follow-up study on the relationship between severity of knee pain and risk of hip fracture in 6641 participants aged over 75 years [23], our study found an overall higher risk of incident fractures in Class 1 and Class 2 compared to Class 3. Similarly, Tatsuhiko et al found an association between the presence of back pain and vertebral fractures in 818 postmenopausal women with a mean 5.7-year follow-up [44]. In a community-based US cohort study with a mean 6.6-year follow-up, the presence of pain symptoms in knee OA had increased risk of non-vertebral fractures in 288 men aged over 70 years [45]. These findings reflect that experiencing pain may be a risk factor for fractures. In contrast, data from the MrOS cohort including 5993 community-dwelling men aged over 65 years reported no association between presence of knee pain and hip and non-spine fractures [24]. This discrepancy might be due to different study designs, pain assessment, and fracture sites assessed.

The current study further showed that associations between pain phenotypes and risk of incident fractures varied depending on fracture sites. The knee pain population classified with most pronounced emotional problems had a relatively higher risk of vertebral than non-vertebral fracture. Few studies have reported associations between psychological problems and vertebral and non-vertebral fracture. In a Danish cohort study of 4114 participants with a mean 7.8-year follow-up period, post-traumatic stress disorder was found to be associated with a higher incidence rate of spine and pelvis fractures than non-vertebral fractures, e.g., hand and wrist, and femur [46]. Similarly, a 13-year follow-up cohort study reported depressive disorders increased the risk of a subsequent new-onset vertebral fracture in adults aged ≥50 years [47]. One possible mechanism underlying the link of pain-related psychological factors and increased risk of fractures, particularly vertebral fracture, may be mediated through inflammatory response and stress hormones such as glucocorticoids, catecholamines, vitamin D [48]. Another possible mechanism is that pain-related psychological factors affect physical function, thereby leading to an increased risk of fractures [49]. In addition, participants classified with structural damages were more likely to have a higher risk of non-vertebral fractures, the reason for this is unclear. Taken together our findings suggest that a specific knee pain phenotype has a predisposition to fractures at a specific site.Targeted prevention for fractures at different sites may be beneficial for a specific pain phenotype. 

The strengths of this study are a large sample and an extended follow-up period of 10.7 years. However, there are several limitations in this study. Firstly, self-reported fractures in this study without X-ray confirmation may have led to an overreporting of fractures [50]. Secondly, there were only three incident hip fracture during follow-ups. Thus, the risk of hip fracture across pain phenotypes cannot be estimated in this study. 

In conclusion, Class 1 and/or Class 2 had a higher risk of incident fractures and falls risk score than Class 3, highlighting that targeted preventive strategies for fractures and falls are needed in pain population.

## Figures and Tables

**Table 1:** Characteristics of the participants by three pain phenotypes.

	Class 1	Class 2	Class 3	*p*-Value		
	(*n* = 345)	(*n* = 157)	(*n* = 413)			
				C1 vs. C2	C1 vs. C3	C2 vs. C3
Age (years)	62.2 (7.5)	64.0 (7.0)	62.6 (7.3)	**0.01**	0.44	**0.047**
Female sex, *n* (%)	242 (70)	44 (28)	179 (43)	**<0.001**	**<0.001**	**0.001**
BMI (kg/m^2^)	28.4 (5.1)	29.3 (4.5)	26.4 (3.9)	**0.04**	**<0.001**	**<0.001**
WOMAC pain score (0–45)	6.2 (7.3)	3.8 (6.3)	1.0 (2.1)	**0.001**	**<0.001**	**<0.001**
Number of painful sites (0–7)	5.0 (1.5)	2.7 (1.7)	1.8 (1.6)	**<0.001**	**<0.001**	**<0.001**
Hip BMD (g/cm^3^)	0.95 (0.14)	1.02 (0.16)	0.96 (0.15)	**<0.001**	0.18	**<0.001**
Falls risk score (zscore)	0.26 (0.90)	0.05 (0.81)	0.10 (0.76)	**0.009**	**0.007**	0.56
Incident fractures from baseline to phase 4, *n* (%)						
Hip fracture	1 (0.3)	1 (0.6)	1 (0.2)	0.53	0.91	0.44
Vertebral fracture	10 (3)	5 (3)	4 (1)	0.84	0.10	0.06
Nonvertebral fracture	58 (17)	19 (12)	44 (11)	0.15	**0.01**	0.65
Any site fracture	66 (19)	24 (15)	48 (12)	0.26	**0.04**	0.27

Values are presented as mean (stand deviation) unless stated otherwise. BMI: body mass index; BMD: bone mineral density; C: Class; WOMAC: Western Ontario and McMaster Universities Arthritis Index. *p*-values are from post hoc testing for comparisons between classes determined by analysis of variance or logistic regression as appropriate. Bold denotes statistical significance.

**Table 2:** The differences in falls risk scores over 10.7-year follow-up and changes in falls risk score among three pain phenotypes.

	Zscore over 10.7-Year Follow-Up β * (95% CI)	Change in Zscore from Baseline to 10.7-Year Follow-Up β ^†^ (95% CI)
Class 1 vs. Class 3	**0.16 (0.09, 0.23)**	**0.03 (0.01, 0.04)**
Class 2 vs. Class 3	**−0.09 (−0.18, −0.002)**	0.02 (−0.003, 0.04)
Class 1 vs. Class 2	**0.25 (0.15, 0.34)**	0.006 (−0.02, 0.03)

* Mixed-effects model including fixed effects for age, physical activity, smoking history, pain medication use at baseline, and random intercepts for follow-ups. ^†^ The interaction terms of pain phenotypes and follow-ups. Bold denotes statistical significance.

**Table 3:** The differences in incident fracture risks over 10.7-year follow-up among three pain phenotypes.

	Incident Fractures over 10.7-Year Follow-Up		
	Vertebral RR * (95% CI)	Non-Vertebral RR * (95% CI)	Any Site RR * (95% CI)
Class 1 vs. Class 3	**2.44 (1.22, 4.91)**	**1.20 (1.01, 1.42)**	**1.24 (1.04, 1.46)**
Class 2 vs.Class 3	1.47 (0.64, 3.36)	**1.41 (1.17, 1.71)**	**1.44 (1.20, 1.73)**
Class 1 vs. Class 2	1.66 (0.73, 3.82)	0.85 (0.70, 1.04)	0.86 (0.71, 1.04)

* Generalized estimating equations log-binomial model included age, physical activity, smoking history, pain medication use, falls risk score, and hip BMD at baseline. Bold denotes statistical significance.

## Data Availability

The datasets generated during and/or analyzed during the current study are available from the corresponding author on reasonable request.

## References

[1] Lehti T.E., Rinkinen M.O., Aalto U., Roitto H.M., Knuutila M., Öhman H., Kautiainen H., Karppinen H., Tilvis R., Strandberg T. (2021). Prevalence of Musculoskeletal Pain and Analgesic Treatment Among Community-Dwelling Older Adults: Changes from 1999 to 2019. Drugs Aging.

[2] Karttunen N.M., Turunen J.H., Ahonen R.S., Hartikainen S.A. (2015). Persistence of noncancer-related musculoskeletal chronic pain among community-dwelling older people: A population-based longitudinal study in Finland. Clin. J. Pain.

[3] Disease G.B.D., Injury I., Prevalence C. (2017). Global, regional, and national incidence, prevalence, and years lived with disability for 328 diseases and injuries for 195 countries, 1990–2016: A systematic analysis for the Global Burden of Disease Study 2016. Lancet.

[4] Briggs A.M., Cross M.J., Hoy D.G., Sànchez-Riera L., Blyth F.M., Woolf A.D., March L. (2016). Musculoskeletal Health Conditions Represent a Global Threat to Healthy Aging: A Report for the 2015 World Health Organization World Report on Ageing and Health. Gerontologist.

[5] Blyth F.M., Briggs A.M., Schneider C.H., Hoy D.G., March L.M. (2019). The Global Burden of Musculoskeletal Pain-Where to from Here?. Am. J. Public Health.

[6] Kittelson A.J., George S.Z., Maluf K.S., Stevens-Lapsley J.E. (2014). Future directions in painful knee osteoarthritis: Harnessing complexity in a heterogeneous population. Phys. Ther..

[7] Butler S. (2012). The impact of chronic pain—European patients’ perspective over 12 months. Scand. J. Pain.

[8] Meisingset I., Vasseljen O., Vøllestad N.K., Robinson H.S., Woodhouse A., Engebretsen K.B., Glette M., Øverås C.K., Nordstoga A.L., Evensen K.A.I. (2020). Novel approach towards musculoskeletal phenotypes. Eur. J. Pain.

[9] ten Klooster P.M., de Graaf N., Vonkeman H.E. (2019). Association between pain phenotype and disease activity in rheumatoid arthritis patients: A non-interventional, longitudinal cohort study. Arthritis Res. Ther..

[10] Smith D., Wilkie R., Croft P., McBeth J. (2018). Pain and Mortality in Older Adults: The Influence of Pain Phenotype. Arthritis Care Res..

[11] Center J.R., Bliuc D., Nguyen T.V., Eisman J.A. (2007). Risk of subsequent fracture after low-trauma fracture in men and women. JAMA.

[12] Center J.R., Nguyen T.V., Schneider D., Sambrook P.N., Eisman J.A. (1999). Mortality after all major types of osteoporotic fracture in men and women: An observational study. Lancet.

[13] Klotzbuecher C.M., Ross P.D., Landsman P.B., Abbott T.A., Berger M. (2000). Patients with prior fractures have an increased risk of future fractures: A summary of the literature and statistical synthesis. J. Bone Miner. Res..

[14] Stone K.L., Seeley D.G., Lui L.Y., Cauley J.A., Ensrud K., Browner W.S., Nevitt M.C., Cummings S.R., Osteoporotic Fractures Research G. (2003). BMD at multiple sites and risk of fracture of multiple types: Long-term results from the Study of Osteoporotic Fractures. J. Bone Miner. Res..

[15] Vranken L., Wyers C.E., van den Bergh J.P.W., Geusens P. (2017). The Phenotype of Patients with a Recent Fracture: A Literature Survey of the Fracture Liaison Service. Calcif. Tissue Int..

[16] Dore A.L., Golightly Y.M., Mercer V.S., Shi X.A., Renner J.B., Jordan J.M., Nelson A.E. (2015). Lower-extremity osteoarthritis and the risk of falls in a community-based longitudinal study of adults with and without osteoarthritis. Arthritis Care Res. (Hoboken).

[17] Gale C.R., Westbury L.D., Cooper C., Dennison E.M. (2018). Risk factors for incident falls in older men and women: The English longitudinal study of ageing. BMC Geriatr..

[18] Leveille S.G., Jones R.N., Kiely D.K., Hausdorff J.M., Shmerling R.H., Guralnik J.M., Kiel D.P., Lipsitz L.A., Bean J.F. (2009). Chronic musculoskeletal pain and the occurrence of falls in an older population. JAMA.

[19] Welsh V.K., Clarson L.E., Mallen C.D., McBeth J. (2019). Multisite pain and self-reported falls in older people: Systematic review and meta-analysis. Arthritis Res. Ther..

[20] Cauley J.A., Barbour K.E., Harrison S.L., Cloonan Y.K., Danielson M.E., Ensrud K.E., Fink H.A., Orwoll E.S., Boudreau R. (2016). Inflammatory Markers and the Risk of Hip and Vertebral Fractures in Men: The Osteoporotic Fractures in Men (MrOS). J. Bone Miner. Res..

[21] Eriksson A.L., Movérare-Skrtic S., Ljunggren Ö., Karlsson M., Mellström D., Ohlsson C. (2014). High-Sensitivity CRP Is an Independent Risk Factor for All Fractures and Vertebral Fractures in Elderly Men: The MrOS Sweden Study. J. Bone Miner. Res..

[22] Pan F., Tian J., Aitken D., Cicuttini F., Jones G. (2019). Pain at multiple sites is associated with prevalent and incident fractures in older adults. J. Bone Miner. Res..

[23] Arden N.K., Crozier S., Smith H., Anderson F., Edwards C., Raphael H., Cooper C. (2006). Knee pain, knee osteoarthritis, and the risk of fracture. Arthritis Care Res..

[24] Munch T., Harrison S.L., Barrett-Connor E., Lane N.E., Nevitt M.C., Schousboe J.T., Stefanick M., Cawthon P.M. (2015). Pain and falls and fractures in community-dwelling older men. Age Ageing.

[25] Pan F., Tian J., Munugoda I.P., Graves S., Lorimer M., Cicuttini F., Jones G. (2020). Do Knee Pain Phenotypes Have Different Risks of Total Knee Replacement?. J. Clin. Med..

[26] Pan F., Tian J., Cicuttini F., Jones G., Aitken D. (2019). Differentiating knee pain phenotypes in older adults: A prospective cohort study. Rheumatology.

[27] Doré D.A., Winzenberg T.M., Ding C., Otahal P., Pelletier J.-P., Martel-Pelletier J., Cicuttini F.M., Jones G. (2013). The association between objectively measured physical activity and knee structural change using MRI. Ann. Rheum. Dis..

[28] Pan F., Laslett L., Blizzard L., Cicuttini F., Winzenberg T., Ding C., Jones G. (2017). Associations Between Fat Mass and Multisite Pain: A Five-Year Longitudinal Study. Arthritis Care Res..

[29] Hoogeboom T.J., den Broeder A.A., Swierstra B.A., de Bie R.A., van den Ende C.H. (2012). Joint-pain comorbidity, health status, and medication use in hip and knee osteoarthritis: A cross-sectional study. Arthritis Care Res..

[30] Knoop J., van der Leeden M., Thorstensson C.A., Roorda L.D., Lems W.F., Knol D.L., Steultjens M.P., Dekker J. (2011). Identification of phenotypes with different clinical outcomes in knee osteoarthritis: Data from the Osteoarthritis Initiative. Arthritis Care Res..

[31] Lord S.R., Menz H.B., Tiedemann A. (2003). A physiological profile approach to falls risk assessment and prevention. Phys. Ther..

[32] Scott D., Blizzard L., Fell J., Jones G. (2009). Ambulatory activity, body composition, and lower-limb muscle strength in older adults. Med. Sci. Sport. Exerc..

[33] Scott D., Hayes A., Sanders K.M., Aitken D., Ebeling P.R., Jones G. (2014). Operational definitions of sarcopenia and their associations with 5-year changes in falls risk in community-dwelling middle-aged and older adults. Osteoporos. Int..

[34] Lanza S.T., Dziak J.J., Huang L., Wagner A.T., Collins L.M. (2015). LCA Stata Plugin Users’ Guide (Version 1.2).

[35] Stubbs B., Schofield P., Binnekade T., Patchay S., Sepehry A., Eggermont L. (2014). Pain is associated with recurrent falls in community-dwelling older adults: Evidence from a systematic review and meta-analysis. Pain Med..

[36] Stubbs B., Binnekade T., Eggermont L., Sepehry A.A., Patchay S., Schofield P. (2014). Pain and the risk for falls in community-dwelling older adults: Systematic review and meta-analysis. Arch. Phys. Med. Rehabil..

[37] Marshall L.M., Litwack-Harrison S., Makris U.E., Kado D.M., Cawthon P.M., Deyo R.A., Carlson N.L., Nevitt M.C., Osteoporotic Fractures in Men Study (MrOS) Research Group (2017). A Prospective Study of Back Pain and Risk of Falls Among Older Community-dwelling Men. J. Gerontol. A Biol. Sci. Med. Sci..

[38] Marshall L.M., Litwack-Harrison S., Cawthon P.M., Kado D.M., Deyo R.A., Makris U.E., Carlson H.L., Nevitt M.C., Study of Osteoporotic Fractures (SOF) Research Group (2016). A Prospective Study of Back Pain and Risk of Falls among Older Community-dwelling Women. J. Gerontol. A Biol. Sci. Med. Sci..

[39] Kitayuguchi J., Kamada M., Inoue S., Kamioka H., Abe T., Okada S., Mutoh Y. (2017). Association of low back and knee pain with falls in Japanese community-dwelling older adults: A 3-year prospective cohort study. Geriatr. Gerontol. Int..

[40] Cai Y., Leveille S.G., Shi L., Chen P., You T. (2022). Chronic pain and circumstances of falls in community-living older adults: An exploratory study. Age Ageing.

[41] Awale A., Hagedorn T.J., Dufour A.B., Menz H.B., Casey V.A., Hannan M.T. (2017). Foot Function, Foot Pain, and Falls in Older Adults: The Framingham Foot Study. Gerontology.

[42] Hicks C., Levinger P., Menant J.C., Lord S.R., Sachdev P.S., Brodaty H., Sturnieks D.L. (2020). Reduced strength, poor balance and concern about falls mediate the relationship between knee pain and fall risk in older people. BMC Geriatr..

[43] Hadjistavropoulos T., Delbaere K., Sherrington C., Lord S.R., Naganathan V. (2021). The Psychology of Fall Risk: Fear, Anxiety, Depression, and Balance Confidence. Falls in Older People: Risk Factors, Strategies for Prevention and Implications for Practice.

[44] Kuroda T., Shiraki M., Tanaka S., Shiraki Y., Narusawa K., Nakamura T. (2009). The relationship between back pain and future vertebral fracture in postmenopausal women. Spine.

[45] Barbour K.E., Sagawa N., Boudreau R.M., Winger M.E., Cauley J.A., Nevitt M.C., Fujii T., Patel K.V., Strotmeyer E.S. (2019). Knee Osteoarthritis and the Risk of Medically Treated Injurious Falls Among Older Adults: A Community-Based US Cohort Study. Arthritis Care Res..

[46] Yuan S., Chen J., Zeng L., Zhou C., Yu S., Fang L. (2021). Association of bone mineral density and depression in different bone sites and ages: A meta-analysis. Food Sci. Nutr..

[47] Lee S.C., Hu L.Y., Huang M.W., Shen C.C., Huang W.L., Lu T., Hsu C.L., Pan C.C. (2017). Risk of Vertebral Fracture in Patients Diagnosed with a Depressive Disorder: A Nationwide Population-Based Cohort Study. Clinics.

[48] Kelly R.R., McDonald L.T., Jensen N.R., Sidles S.J., LaRue A.C. (2019). Impacts of Psychological Stress on Osteoporosis: Clinical Implications and Treatment Interactions. Front. Psychiatry.

[49] Talaei-Khoei M., Fischerauer S.F., Jha R., Ring D., Chen N., Vranceanu A.-M. (2018). Bidirectional mediation of depression and pain intensity on their associations with upper extremity physical function. J. Behav. Med..

[50] Ivers R.Q., Cumming R.G., Mitchell P., Peduto A.J. (2002). The accuracy of self-reported fractures in older people. J. Clin. Epidemiol..

